# HMA-YOLO: a high precision and lightweight detection model of corn trumpet in corn precision pesticide application system

**DOI:** 10.3389/fpls.2026.1785800

**Published:** 2026-03-05

**Authors:** Chengxiang Zhang, Wenqiang Li, Lili Wu, Yuqing Xing, Xueli Qi

**Affiliations:** 1 College of Science, Henan Agricultural University, Zhengzhou, China; 2 Institute of Molecular Breeding, Henan Academy of Agricultural Sciences, Zhengzhou, China

**Keywords:** corn trumpet, lightweight, NVIDIA Jetson Xavier NX, object detection, precision pesticide application

## Abstract

**Introduction:**

Pests and diseases significantly reduce the quality and yield of corn, while the corn precision pesticide application system is one of the effective measures to solve this problem. However, the detection of corn trumpets in complex farmland environments poses significant challenges due to the high color similarity between corn trumpets and the background, the small target size, and occlusion by corn leaves.

**Methods:**

In this paper, we propose a lightweight HMA-YOLO model to accurately detect corn trumpets in agricultural background based on YOLOv12n model. Firstly, The HCT structure that is based on CNN and Transformer architectures with assignable feature map channels is introduced into the backbone network to extract target feature information and enhance the ability of the model to distinguish between targets and backgrounds. Secondly, an efficient multi-branch and multi-scale feature pyramid network (MBMS-FPN) is developed to enhance the extraction and fusion of deep-level features of targets of varying sizes, which employs the neck heterogeneous kernel selection mechanism and feature weighted fusion module. Finally, an efficient and lightweight asymmetric multi-level channel compression detection head (AMCCDH) is improved to alleviate missed detections caused by occlusion. The AMCCDH improves detection accuracy by deepening the network path of the IoU task branch and expanding its receptive field by using 3×3 depth-wise separable convolutions. Moreover, these three improvement measures all undergo lightweight processing.

**Results and discussion:**

Experimental results show that HMA-YOLO achieves a mAP@0.5 of 91.5%, precision of 89.8%, and recall of 83.7%, operating at 128 FPS with only a model size of 3.1 MB and a parameter count of 1.407M. This model outperforms mainstream object detectors and has been successfully deployed on the NVIDIA Jetson Xavier NX embedded platform, which achieves real-time and efficient detection in resource-constrained environments.

## 1 Introduction

China is a major corn-producing countries in the world. The Ministry of Agriculture and Rural Affairs of the People’s Republic of China pointed out that corn pests and diseases were widespread across the country, affecting an area of 980 million mu, which posed a serious threat to corn quality and yield in 2024. Accordingly, effective prevention and control of corn pests and diseases not only reduces the economic losses but also ensures national food security (Savary et al., 2012; Wang and Chen, 2021; Cui et al., 2024). Although traditional manual or carpet style pesticide spraying can effectively control pests and diseases, it can also cause pesticide waste and environmental pollution. Therefore, precise application of pesticides to corn is the key to solving the above problems (Xu et al., 2025; Qaswar and Mouazen, 2025; Mamabolo et al., 2025). The corn trumpet precision application system has long been a research hotspot in the field of corn precision application solutions. The system operates by precisely identifying the corn trumpet, then driving the motor to perform quantitative pesticide application. This method is considered one of the exemplars of smart agriculture (Sun et al., 2019; Diao et al., 2025), as it not only significantly improves application efficiency but also reduces pesticide usage. In this system, the precise identification of the trumpets is a prerequisite for successful pesticide application.

In recent years, advances in computer vision have provided new ideas and solutions for the task of accurately detecting corn trumpets. Computer vision technology can significantly improve the accuracy in identifying the trumpets. Object detection models based on deep learning (DL), such as convolutional neural networks (CNN), first extract features from images and then identify objects in the images (Xiao et al., 2023). CNN-based object detection models are broadly categorized into two-stage and one-stage methods. Two-stage algorithms such as R-CNN (Girshick et al., 2014), Faster R-CNN (Ren et al., 2017), and Mask-RCNN (He et al., 2017) improve object detection accuracy by optimizing region proposal mechanisms and feature extraction networks. These methods have been successfully applied to detect specific parts of plants in complex environments. Zhang et al. (2022) proposed an improved Faster R-CNN for rice panicles identification and achieved the mean average precision (mAP) of 92.47%. Wang et al. (2023) optimized the Mask-RCNN to detect tea picking points with a mean average precision of 93.95% and a recall of 92.48% in complex environments. However, while the high detection accuracy for two-stage methods, the computational intensity of two-stage models limits their applicability in real-time, resource-constrained field scenarios.

In contrast, to improve detection speed and ensure real-time detection, researchers proposed a single-stage object detection algorithm. Representative single-stage object detection algorithms includes SSD (Liu et al., 2016) and the YOLO (Redmon et al., 2016) series algorithms. Sun et al. (2021) detected apple pests and diseases based on an improved SSD model, resulting in a detection speed of 12.53 FPS while a mAP of 83.1%. Upadhyay et al. (2025) distinguished weeds and crops by using YOLOv11 model, the inference time is just 10ms, whereas the mAP is only 81.9%. Liu et al. (2024) proposed a lightweight scale-adaptive network (SANet) for salient object detection by integrating the SAFE and MFA modules, which achieves a trade-off between lightweight restriction and detection performance. Liu et al. (2025a) proposed a lightweight CMR U-Net detection model by integrating the multi-directional attention module and contour loss function, which achieves superior detection performance. In order to further improve accuracy, a large number of scholars have made a series of improvements based on YOLO series algorithms. Hu et al. (2025) used YOLOv5 as the baseline model and introduced the Conv-SPD network module into the backbone network to replace the original stride convolution for feature extraction, which increases the mAP of 1.1%. Dong et al. (2025b) proposed a lightweight rail defect detection network REDNet based on YOLOv8, which integrates the RevCol backbone, MSDFA module, and ATDH detection head. The network achieved a mean Average Precision (mAP) of 94.1% on the custom dataset, with only 5.70 million parameters and an inference speed of 204.1 FPS, which makes it convenient for subsequent engineering deployment due to its advantages of lightweight design and high real-time performance. Dong et al. (2025a) proposed a lightweight remote sensing small target detection framework RSNet, which embeds an adaptive downsampling module (ADown) and a lightweight hybrid convolution module (GSConv) in the backbone network and designs a compact alignment detection head (CADH). On the NWPU VHR-10 dataset, the mean average precision (mAP) score of RSNet reached 92.2%, and the number of parameters was reduced by 41.9% compared with the baseline model, which achieves a perfect balance between high detection accuracy and computational efficiency. In addition, accurate detection of small targets and occluded targets in certain special scenarios is also a key area of research. Chutichaimaytar et al. (2025) improved feature extraction capabilities of the model by introducing a multi-scale sequence feature fusion (MSFF) module into the YOLOv8n backbone network, resulting in a 2% improvement in mAP. Jiang et al. (2024) proposed a cabbage detection algorithm based on the YOLOv8n neural network (YOLOv8-Cabbage), and constructed a positioning system in combination with a RealSense depth camera, which enables the real-time detection of cabbages in complex field environments and effectively solve the problem of low detection accuracy caused by similar backgrounds and target occlusion. CNN-based feature extraction networks demonstrate excellent performance in local feature extraction. However, the feature extraction methods mainly rely on the use of convolution kernels to integrate and abstract different scale receptive fields. Correspondingly, convolution operations lack the ability to perceive global image information and are unable to model the dependencies between features (Fang et al., 2021).

Recently years, researchers successfully expanded the application of Transformer models from natural language processing to computer vision tasks (Carion et al., 2020). Compared with CNN, the Transformer model adopts an end-to-end design and models the global correlation of the target image, enabling the model to fully utilize image context information for target feature extraction. For example, object detection models based on Transformers, such as Vision Transformer (Dosovitskiy et al., 2020), RT-DETR (Zhao et al., 2024), and Swin Transformer (Liu et al., 2021), have become popular research topics in the field of computer vision. Furthermore, these Transformer-based models have been successfully applied to visual detection tasks related to agriculture. Li et al. (2023) proposed a spatial convolution self-attention Transformer model to extract strawberry features based on the Transformer architecture, which increases the mean average precision (mAP) by 1.02%. By combining the neck network of YOLOv5 with a Vision Transformer, Xu et al. (2024) proposed a high-precision, low-complexity W-YOLOv5 object detection model, where the mAP is enhanced by 4.4% compared with YOLOv5. Futhermore, Liu et al. (2025b) addressed the challenge of accurately detecting small pests in complex real-world rice field environments by introducing a novel Small Target Improvement Pyramid (STIP) neck layer into the DETR model and combining the cross-level partial network concept with a full-kernel module featuring large-kernel convolutions. Although detection accuracy could be improved by the above Transformer-based object detectors, this models still suffer from high computational complexity, slow inference speeds, and insufficient perception capabilities for small objects.

To address these shortcomings, this study proposed HMA-YOLO, a lightweight and efficient object detector specifically designed for detecting corn trumpets. HMA-YOLO proposed several key innovative strategies based on YOLOv12n to deal with the limitations of existing models. These include a lightweight hybrid structure of CNN and Transformer (HCT) to replace the A2C2f modules in the P4 and P5 feature layers of the original backbone network, which enhances the ability of the model to extract global and local features of targets in complex environments. An efficient multi-branch and multi-scale feature pyramid network (MBMS-FPN) was designed to capture small object feature information and improve the adaptability to height variations of the corn trumpets during detection. Additionally, an efficient and lightweight asymmetric multi-level channel compression detection head (AMCCDH) was introduced to address the issue of missed detections caused by structural occlusions of corn leaves. These improvement strategies enable HMA-YOLO to not only effectively address the challenges of the corn trumpets detection tasks, but also meet the requirements of lightweight deployment.

**Figure 1:**
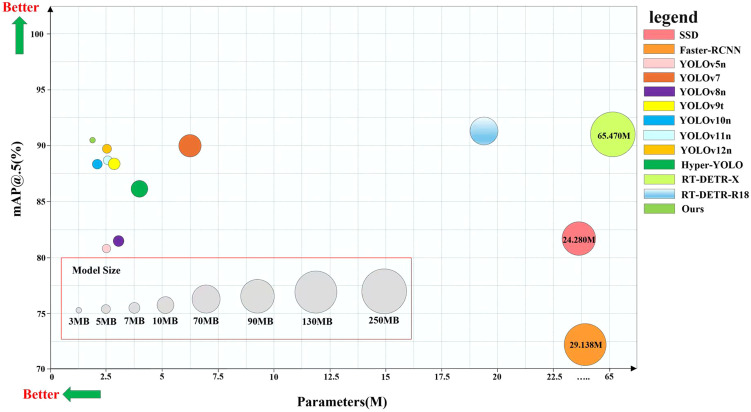
Comparison of mAP@0.5, model parameters and model size for different algorithms.

The main contributions of this paper are as follows:

1. We constructed a rich dataset covering three key states of corn trumpets: complex backgrounds (highly similar to background colors), small sizes (including corn trumpets of different heights), and varying degrees of structural occlusion caused by corn leaves. Furthermore, this dataset underwent comprehensive data augmentation to serve as detection resources for improving the model.
2. Three model optimization strategies were implemented on the YOLOv12n network to improve the accuracy and efficiency of corn trumpets detection. The HCT structure was first introduced into the backbone network to enhance the ability of the model to extract target features in complex backgrounds. Next, the MBMS-FPN structure, which was designed to integrate a weighted feature fusion module and a neck heterogeneous kernel selection mechanism utilizing depth-wise convolution based on multi-scale convolutional kernel, enables the model to effectively capture small target features while further enhancing its adaptability to height variations during trumpet detection. Finally, the detection robustness of the network in occluded scenarios was significantly improved by the introduction of AMCCDH. HMA-YOLO, a high-precision lightweight object detector that we proposed, achieves a mAP@0.5 of 91.5%, an accuracy of 89.8%, and a recall of 83.7%, with an FPS of 126.9 s^-1^. Notably, parameter count, computational complexity, and model size of our model are only 1.407M, 5.1G, and 3.1MB, respectively. Compared to mainstream object detectors, as shown in **Figure 1**, HMA-YOLO demonstrates superior performance in terms of overall performance.
3. We used NVIDIA Jetson Xavier NX, an edge computing device, to deploy HMA-YOLO, thereby verifying that our proposed HMA-YOLO can not only detect corn trumpets quickly and accurately, but also meet the requirements for deployment on embedded platforms with limited computing resources.

## 2 Materials and methods

**Figure 2:**
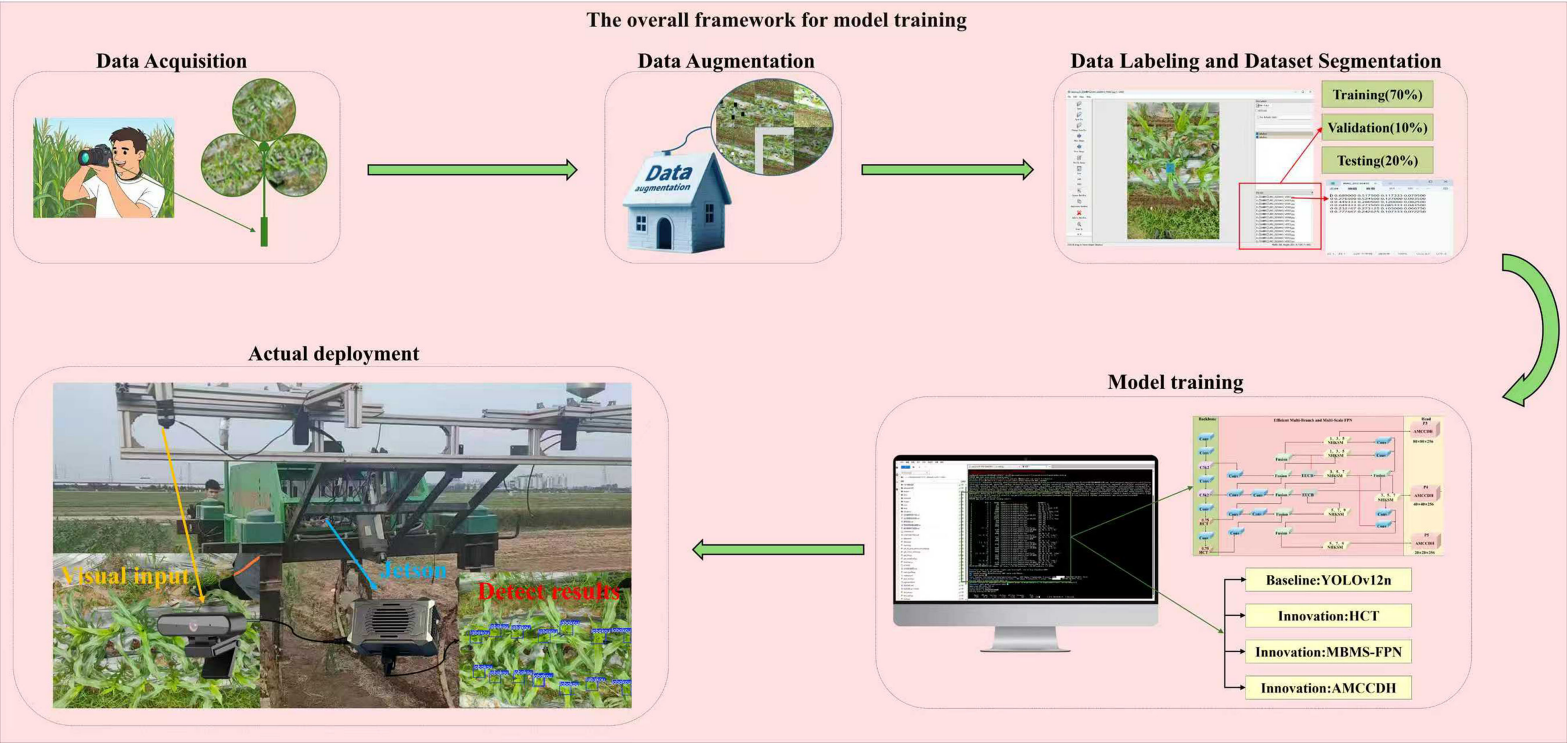
The overall framework of the model training process, including data acquisition, data preprocessing, model training, and model deployment.

As shown in **Figure 2**, this study constructed an overall framework for model training, which uses a flowchart to intuitively illustrate the entire process from data preparation to model deployment.

### 2.1 Data acquisition

**Figure 3:**
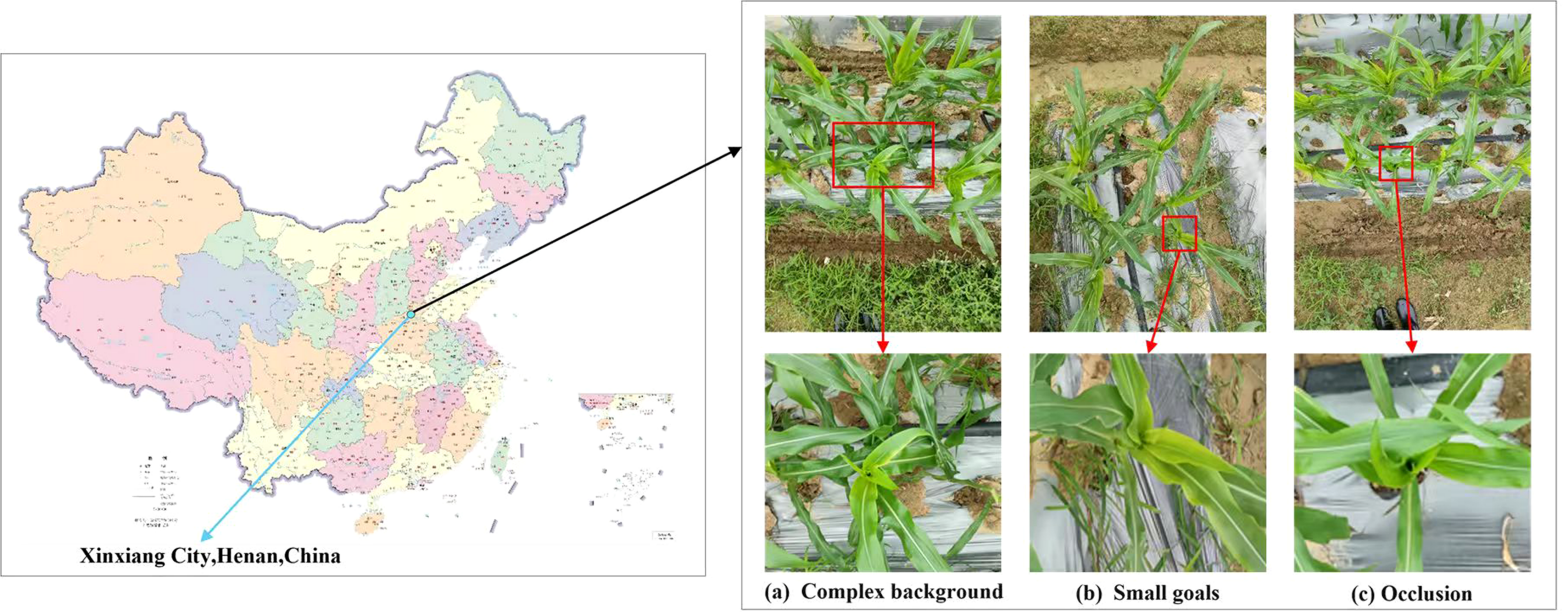
Information on the location of data collection and Example image of data characteristics: **(a)** Complex background; **(b)** Small goals; **(c)** Occlusion.

The dataset for this study was obtained from the 207th experimental field of Henan Agricultural University in Xinxiang (35.1724^°^N, 113.8113^°^E). We used a high-resolution camera to capture images of corn trumpets under different conditions, which ensures comprehensive coverage of the following key issues: significant similarity between the target and background, target size at a relatively small pixel scale, and the impact of structural occlusion caused by corn leaves. Examples of these issues are shown in **Figure 3**. The camera position was not fixed and captured images from various overhead angles. Specifically, the camera was positioned within a range of 0.2 meters to 1.0 meters from the corn trumpets. After careful selection, a total of 800 high-resolution images were obtained, which have resolutions of 3000×4000 and 4000×3000 pixels, all saved in JPG format.

### 2.2 Data augmentation and labeling

**Figure 4:**
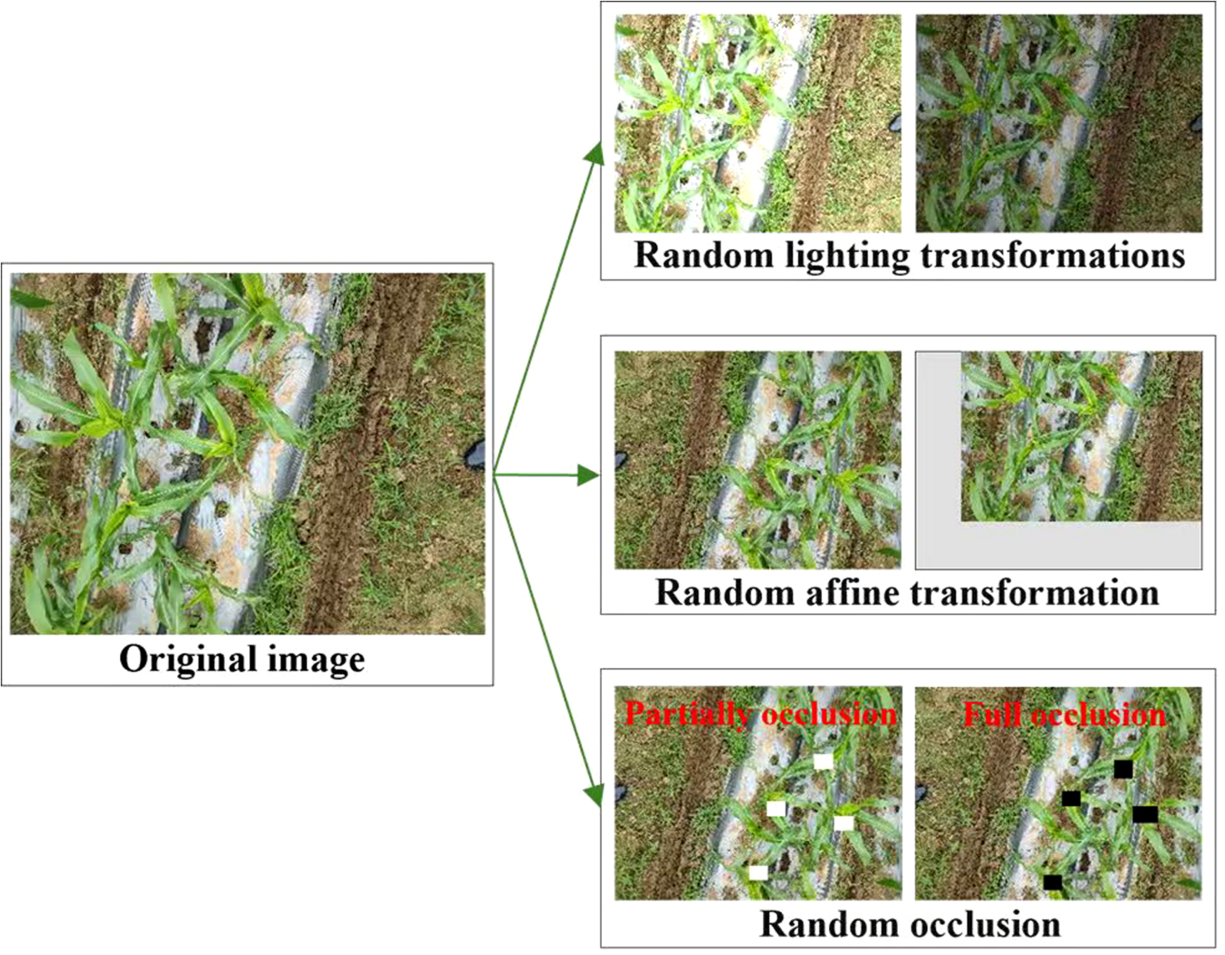
Display of images after data enhancement.

During the data preprocessing stage, we filtered out low-quality images. In order to improve generalization ability of the model, a set of data augmentation techniques were applied, as shown in **Figure 4**, including:

1. Random occlusion: Randomly select the location and size of occlusion areas (full occlusion and partial occlusion) in the original image. This operation simulates random occlusion situations that may occur in real-world scenarios, which helps the model improve its robustness when faced with local occlusion.
2. Random lighting transformations: Simulate natural light changes by increasing or decreasing image brightness. This operation allows the model to better capture the color changes of the detected object under different lighting conditions.
3. Random affine transformations: Randomly rotate and translate images to simulate detection targets from different perspectives. This operation can improve the model’s adaptability to different scale transformations of detection targets, effectively enhancing the stability and accuracy of its prediction performance.

The final dataset was expanded from the original 800 images to 2, 600 images. We used the Labelimg tool to annotate the augmented dataset, with each corn trumpets precisely annotated within a rectangular bounding box. For corn trumpets obscured by corn leaves, the bounding boxes were defined based on experience. We also generated text files containing rectangular bounding box class information and position information, with the text file names matching the corresponding image names. To facilitate model evaluation and training and prevent overfitting, the 2, 600 images after data augmentation were randomly divided into training, testing, and validation sets in a 7:2:1 ratio.

### 2.3 Overall architecture of the HMA-YOLO model

This study focuses on visual detection of corn trumpets in a precision application system. The detection task is conducted in a complex agricultural environment, which places high demands on the accuracy and real-time performance of the model. Compared with other mainstream object detection models, YOLOv12n achieves a satisfactory balance among parameter efficiency, performance, detection accuracy, and real-time performance, which is particularly important for trumpet detection.

**Figure 5:**
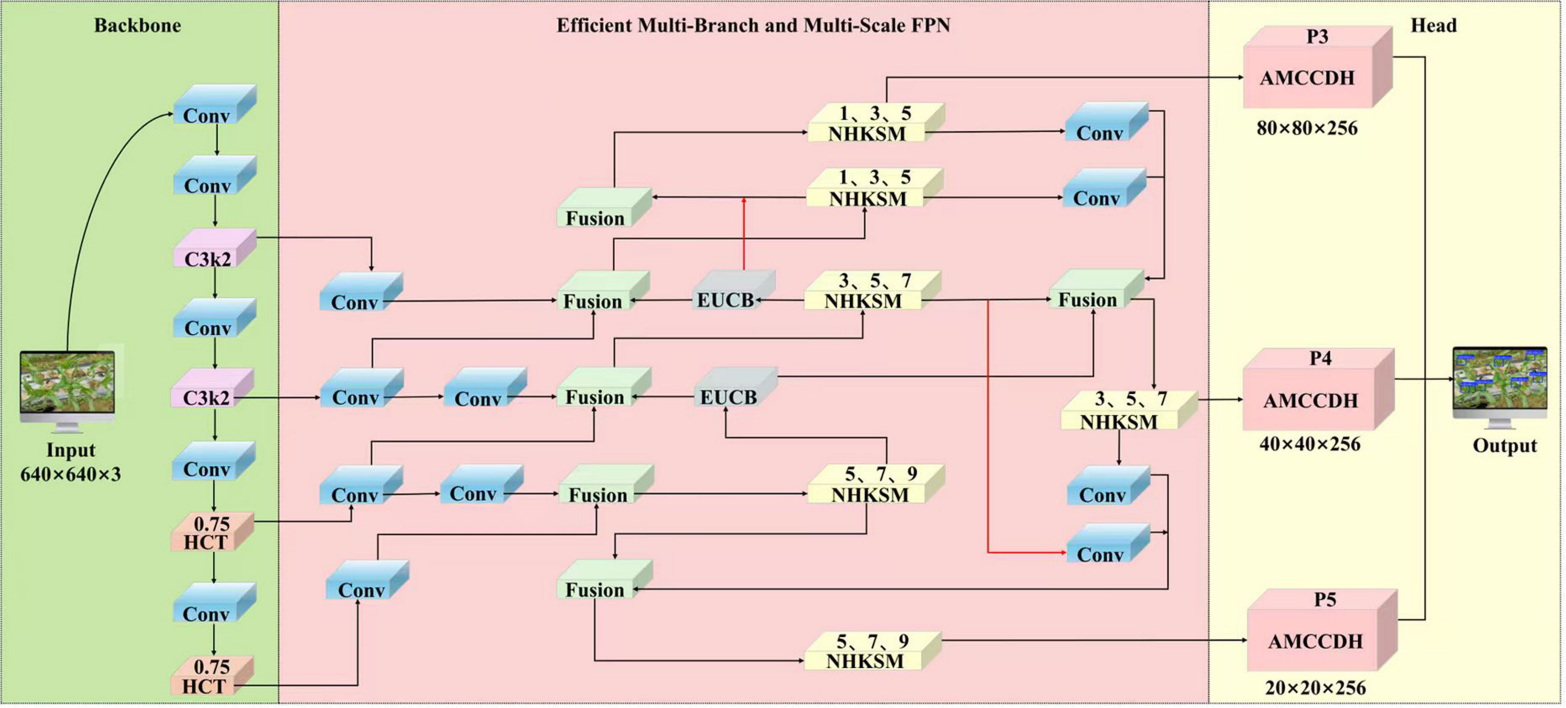
Structure of HMA-YOLO.

The YOLOv12n network consists of a backbone network for feature extraction, a neck network for feature fusion, and a prediction head. Although YOLOv12n could meet common object detection tasks, the model network was still improved in three key aspects considering the characteristics of the corn trumpets dataset as shown in **Figure 5**. (1) A hybrid structure of CNN and Transformer (HCT) was introduced to the P4 and P5 feature layers of the backbone network to replace the regional attention module (A2C2f in YOLOv12n). The HCT structure could dynamically adjust the ratio of input channels between CNN and Transformer to provides a better balance between computational efficiency and feature extraction capabilities. (2) An efficient Multi-Branch and Multi-Scale FPN structure (MBMS-FPN) was proposed to extract deep feature information of small targets and improve the robustness of the model regarding the height variation of the targets. (3) An efficient and lightweight asymmetric multi-level channel compression detection head (AMCCDH) was designed to alleviate the problem of missed detection through deepening the network path of the IoU task branch and using 3×3 depth-separable convolutions.

#### 2.3.1 Hybrid structure of cnn and transformer

**Figure 6:**
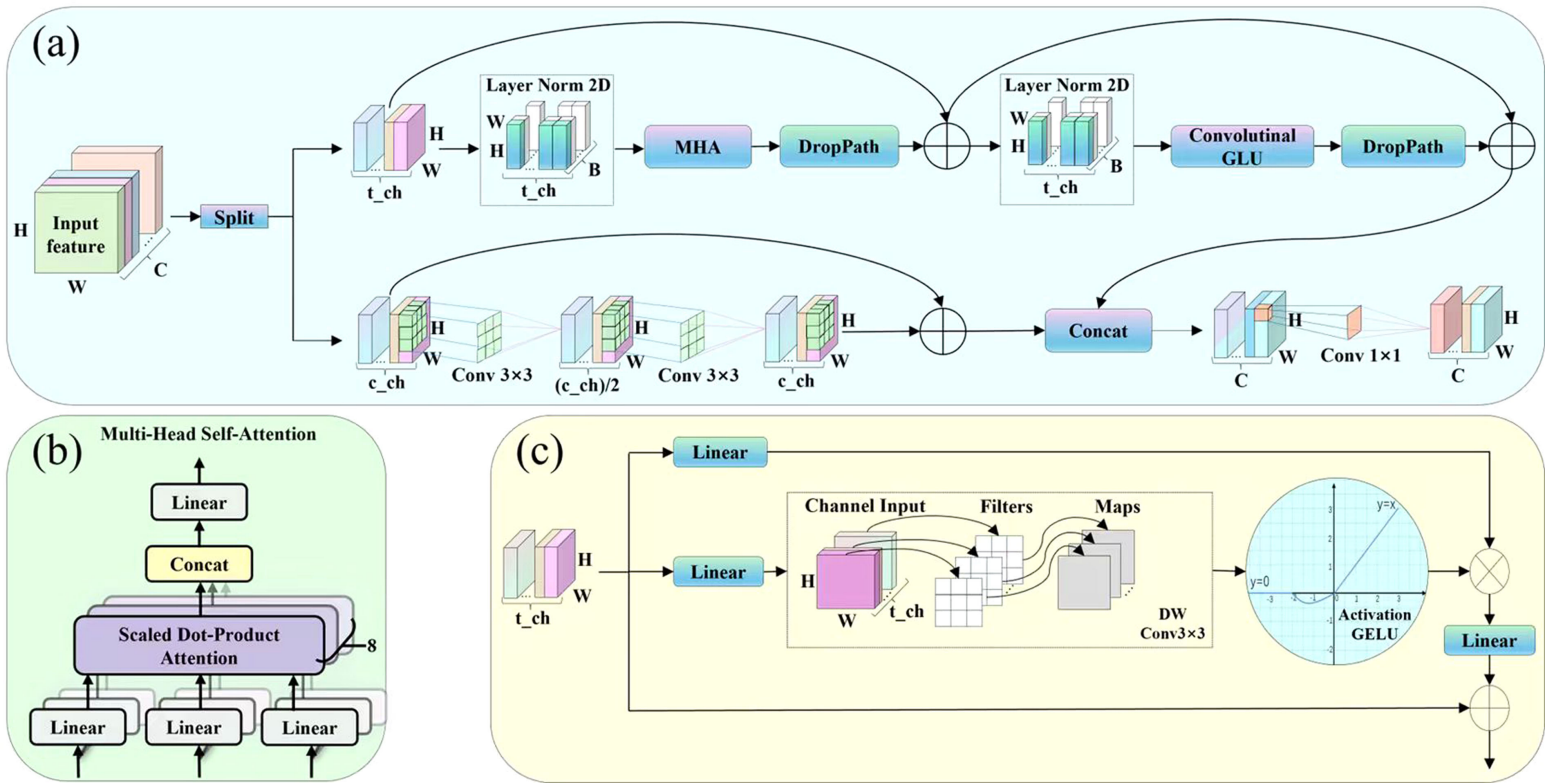
Hybrid structure of cnn and transformer: **(a)** Structure of HCT; **(b)** Structure of MHA; **(c)** Structure of CGLU.

The A2C2f module of YOLOv12 backbone network continues and evolves the idea of convolutional neural networks (CNN). While maintaining the core role of convolution operations in feature extraction, it improves detection performance of the model through improvements such as residual connections, regional attention mechanisms, and multi-scale fusion (Tian et al., 2025). However, it is worth considering that the A2C2f module is still based on the CNN architecture, which has a small field of view when extracting features, can only extract local features, and may thus lead to the loss of target feature information, particularly in complex scenes where the target and background colors are similar. In addition, the high computational complexity of this module increases model parameters and reduces detection speed. To address these issues, a Transformer structure with global feature extraction capabilities was introduced. Given the high computational complexity of this structure, which could lead to a significant increase in computational overhead, the HCT structure was designed to ensure efficient extraction of target features in complex scenes while reducing computational costs, as shown in (**Figure 6a**). This structure first divides the input channels of feature map into two parts in a 0.25: 0.75 ratio, which are processed by CNN and Transformer, respectively (the validity of this allocation ratio will be verified in the 3.4 ablation experiment of HCT structure channel allocation). Subsequently, the processed feature maps are concatenated. Finally, a 1×1 convolution is used for feature fusion and output. The following sections will describe the Transformer and CNN modules of our hybrid structure separately.

Transformer block: This module consists of a multi-head self-attention mechanism (MHA) (Vaswani et al., 2017) and a convolutional gated linear unit (CGLU) (Shi, 2024) that introduces channel attention based on nearest image features. The MHA structure is shown in (**Figure 6b**). First, maps the query, key, and value through eight different linear transformations. Then, the different attentions are concatenated. Finally, another linear transformation is performed to extract global features. The CGLU structure shown in (**Figure 6c**), consists of two linear projections that are multiplied element-wise. One of these projections passes through a 3×3 depth-wise convolution in its smallest form that enables the CGLU structure to comply with the gated channel attention mechanism (Yang et al., 2020) and then activated by the GELU gating function. Compared to Feed-Forward Networks (FFN), this structure achieves the attentionalization of the channel mixer with fewer floating-point operations, while simultaneously enhancing nonlinear feature expression capabilities of the model.

*CNN block*: The CNN module consists of two 3×3 convolutions. Specifically, the feature map first passes through the first 3×3 convolution to reduce the channel dimension, with the aim of extracting local core features (such as texture, edges, etc.). Subsequently, this feature map passes through the second 3×3 convolution to restore the dimension and expand the feature expression capability. Finally, the processed feature map and the original feature map are connected by residual to enhance feature expression and outputs.

#### 2.3.2 Efficient multi-branch and multi-scale FPN

In this section, the efficient MBMS-FPN is proposed, which is designed to process multi-level features that are extracted from the backbone network. MBMS-FPN consists of efficient up-convolutional block (EUCB), fusion block and neck heterogeneous kernel selection mechanism (NHKSM) to improve detection performance for small objects and enhance the adaptability of the model regarding the height variation of the corn trumpets, simultaneously achieving lightweight design. The different modules of MBMS-FPN are described below:

##### 2.3.2.1 Efficient up-convolution block

**Figure 7:**
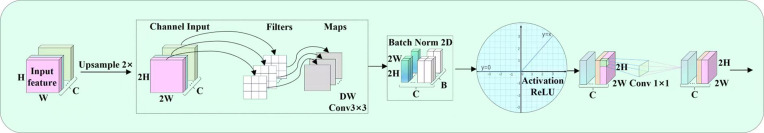
Structure of EUCB.

The upsample module in YOLOv12n only addresses the issue of size matching for feature maps across different levels. However it cannot effectively solve the problem of depth matching for cross-level features in terms of spatial position and detail granularity. Accordingly, an EUCB module was designed to replace upsample module of YOLOv12n as shown in **Figure 7**. The EUCB firstly uses upsampling with an expansion factor of 2 to proportionally expand the resolution of the input feature map. Then, it enhances feature expression capabilities by using 3×3 depth-wise convolution, batch normalization layers (BN), and ReLU activation functions. Finally, a 1×1 convolution is used to match the channel dimension of the next level. In our experiments, it should be noted that the channel dimension of the input and output feature maps for MBMS-FPN structure has been standardized to 256, which aims to reduce the model parameters and maintain detection accuracy. (This will be further elaborated in the 3.5 comparison experiments of MBMS-FPN fixed channel dimension).

##### 2.3.2.2 Fusion block

When fusing features of different sizes, the YOLOv12n model still adopts the conventional approach, which first unifies the feature sizes, after which concatenation operations are performed, and employs global self-attention upsampling to restore pixel location information. However, due to differences in the input feature sizes, the contribution to the output features varies. If the above feature fusion method is used, redundant information will interfere with the model learning of key features, reducing the fusion effect. In this study, a multi-scale feature weighting fusion mechanism was employed to resolve the above problems (Tan et al., 2020), which specifically assigns additional weights to each input feature and allows the network to autonomously learn the importance of each input feature. Based on this idea, we considered rapid normalization fusion: $O=\sum_{i}\frac{\omega_{i}}{\in +\sum_{j}\omega_{j}}\cdot P_{i}$. Taking fusion node 13 (layer 13 of the network structure) as an example. First, the original weight parameters $\omega_{1}$ and $\omega_{2}$ are assigned to input features P4 and P5, respectively, and a ReLU activation function is introduced after each original weight $\omega_{i}$, which ensures that $\omega_{i}\geq 0$. Then, the normalized weights $\frac{\omega_{i}}{\in +\omega_{1}+\omega_{2}}$ are calculated (where *i* = 1, 2, and ∈, which serves as the stability value for normalized weights, is set to $10^{-4}$). Finally, these normalized weights are multiplied element-wise with their corresponding features, after which they are summed to yield the weighted fused features. This fusion method, which is designed to eliminate redundant feature information while retaining key features to improve detection accuracy, also achieves a reduction in model parameters and computational load through a process in which concatenation is replaced with addition.

##### 2.3.2.3 Neck heterogeneous kernel selection mechanism

**Figure 8:**
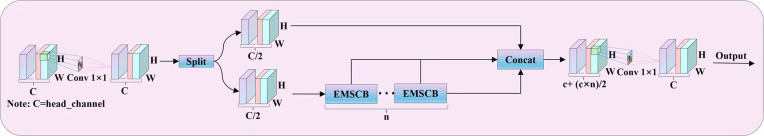
Structure of NHKSM.

From a macro-architectural perspective, a deeper understanding of the use of neck convolutions in YOLOv12n was gained. Fixed-size convolutions, which are employed for feature enhancement and multi-scale feature extraction, are not the optimal choice for extracting multi-scale feature information. This is because convolutions with the same kernel size, which possess a fixed receptive field, cannot adapt to targets of varying sizes. To address this issue, we designed the NHKSM based on the CSP structure, which drew inspiration from the heterogeneous kernel protocol, as shown in **Figure 8** (Chen et al., 2025). This mechanism consists of efficient multi-scale convolutional block (EMSCB) and depth-wise convolution based on multi-scale convolutional kernel (DCMSCK). However, it is worth noting that we used different DCMSCK across different feature layers. This is because the object detector extracts high-resolution features from lower-level feature layers, and applying small convolutional kernel groups to these layers is suitable for detecting small objects. Conversely, higher-level feature layers extract low-resolution features, and applying larger convolutional kernel groups to these layers is suitable for detecting large targets. If we fix DCMSCK across different feature layers, where for instance depth-wise convolutions based on large multi-scale convolutional kernels are applied to lower-level feature layers to detect small targets, it would lead to an increase in the computational overhead of the object detector. Therefore, we opted to employ multi-scale depth-wise convolutions with kernel sizes of 1, 3, and 5 in the low-level P3 feature layer within the neck network of the model; multi-scale depth-wise convolutions with kernel sizes of 3, 5, and 7 in the mid-level P4 feature layer; and multi-scale depth-wise convolutions with kernel sizes of 5, 7, and 9 in the high-level P5 feature layer to accommodate different resolution requirements.

This design significantly improves the detection accuracy of the target detector for small targets such as corn trumpets, and also improves adaptability to different heights of corn trumpets during detection, while complying with the lightweight design concept to reduce model computing overhead. The EMSCB and DCMSCK modules of our NHKSM are described as follows:

###### 2.3.2.3.1 Efficient multi-scale convolutional block

**Figure 9:**
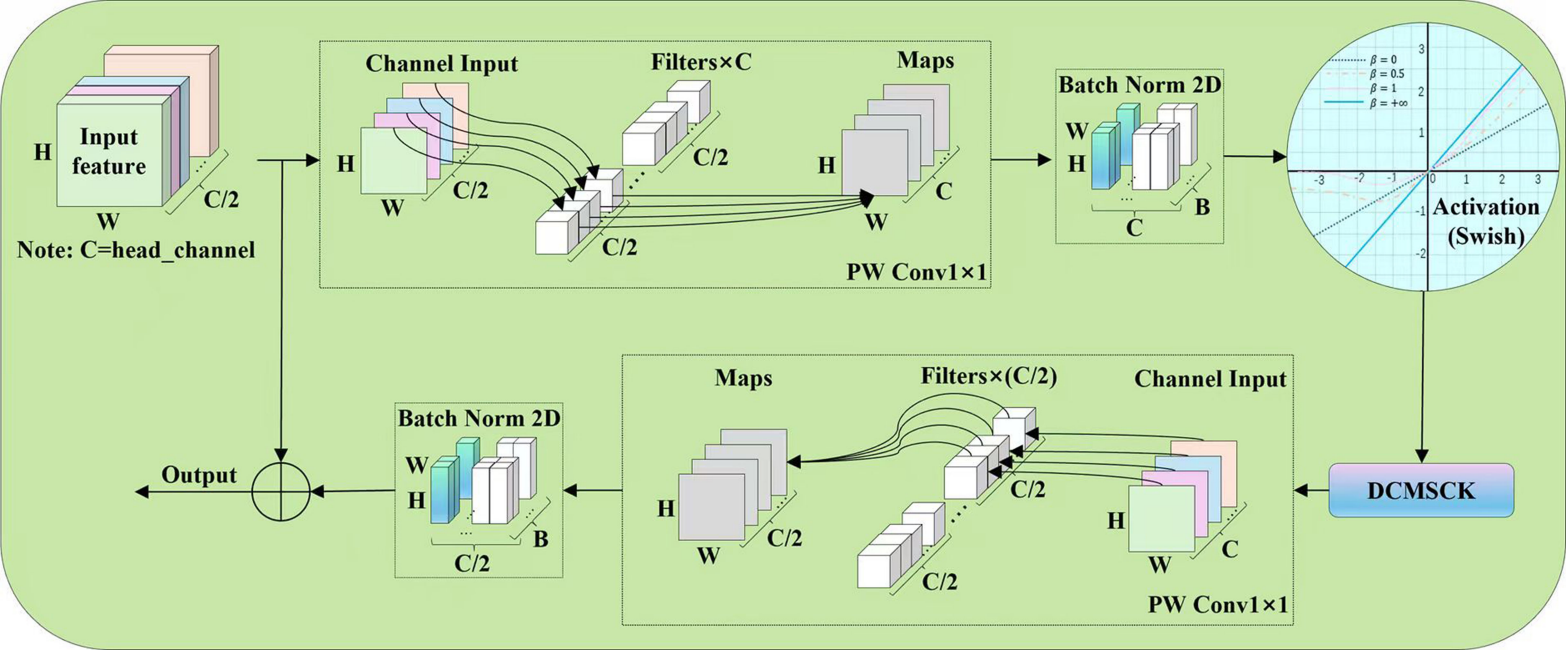
Structure of EMSCB.

We employed an efficient multi-scale convolution module to enhance the features generated by the cascaded expansion path, as shown in **Figure 9**. This block is based on the design concept of the inverse residual block (IRB) in MobileNetV2 (Sandler et al., 2018), but unlike IRB, it uses multi-scale convolutional kernels for depth-wise convolution operations. Specifically, in our EMSC, we first used a 1×1 point-wise convolution with an expansion factor of 2 to expand the channel dimension of the input feature map. Then, after passing through a batch normalization layer (BN) and a ReLU activation function layer, we immediately used a DCMSCK to capture information from the current feature layer. Subsequently, we utilized a 1×1 point-wise convolution and a batch normalization layer (BN) to restore the original channel dimension of the feature map. Finally, we performed a residual connection between the input feature map and the feature map processed by the EMSCB module to enhance feature representation and output the feature map.

###### 2.3.2.3.2 Deep-wise convolution based on multi-scale convolutional kernel

**Figure 10:**
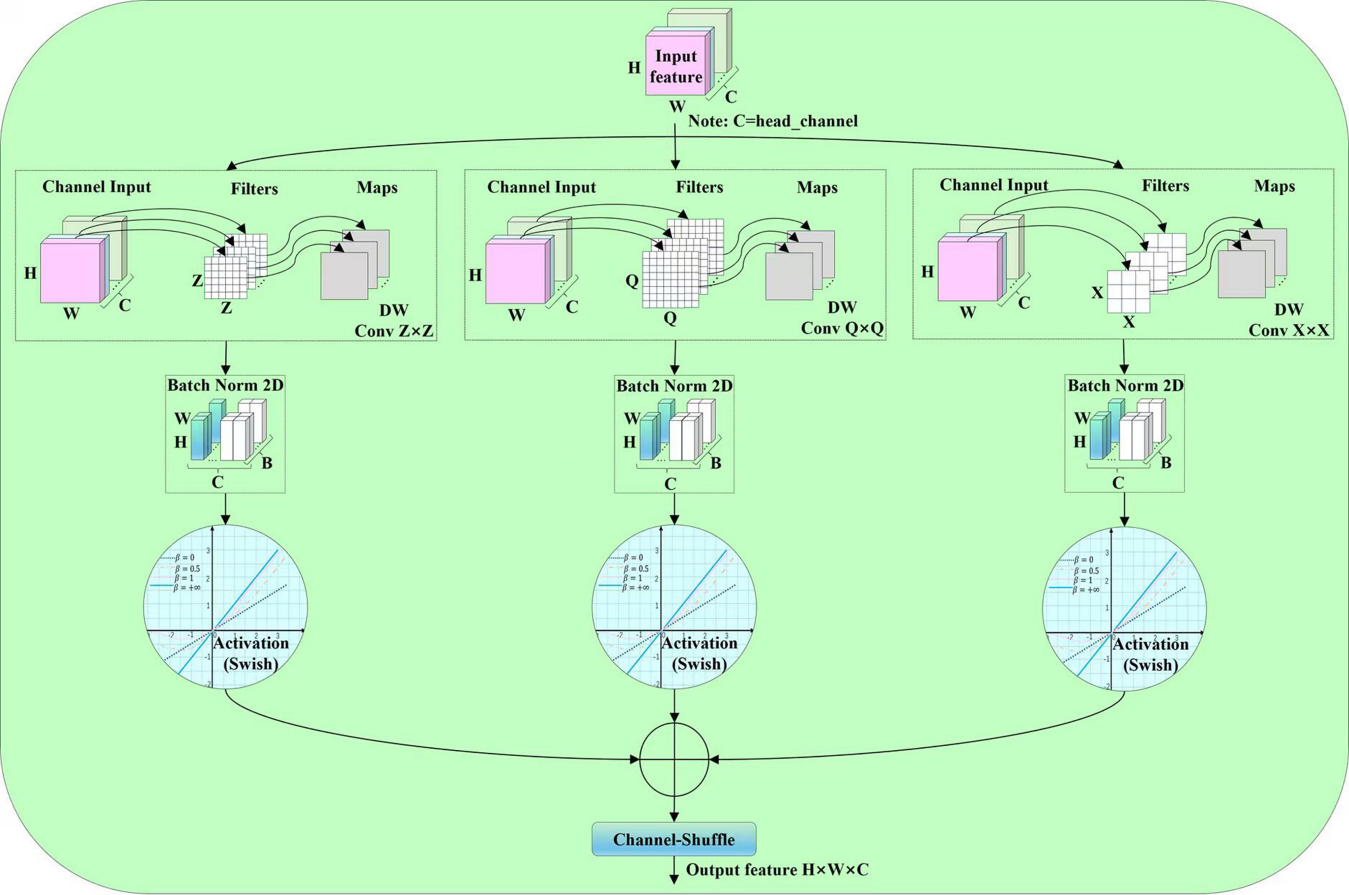
Structure of DCMSCK.

A set of multi-scale depth-wise convolutions is used to extract information from the current feature layer. The DCMSCK structure shown in **Figure 10**, first performs depth-wise convolution operations in parallel across multiple scales on the input feature map. However, it is worth noting that the parallel convolutional structure for feature extraction can effectively reduce parameter redundancy (its effectiveness will be validated in the 3.6 comparison experiment on the size of the neck heterogeneous kernel group). Then, the feature maps processed by depth-wise convolutions at each scale are subjected to processing through a batch normalization layer (BN) and a ReLU activation function layer. Subsequently, the feature maps undergo a residual connection. Finally, a channel shuffler (Zhou et al., 2018) is used to shuffle channels across groups to restore inter-channel relationships, and the feature map is output.

#### 2.3.3 Efficient and lightweight asymmetric multi-level channel compression detect head

**Figure 11:**
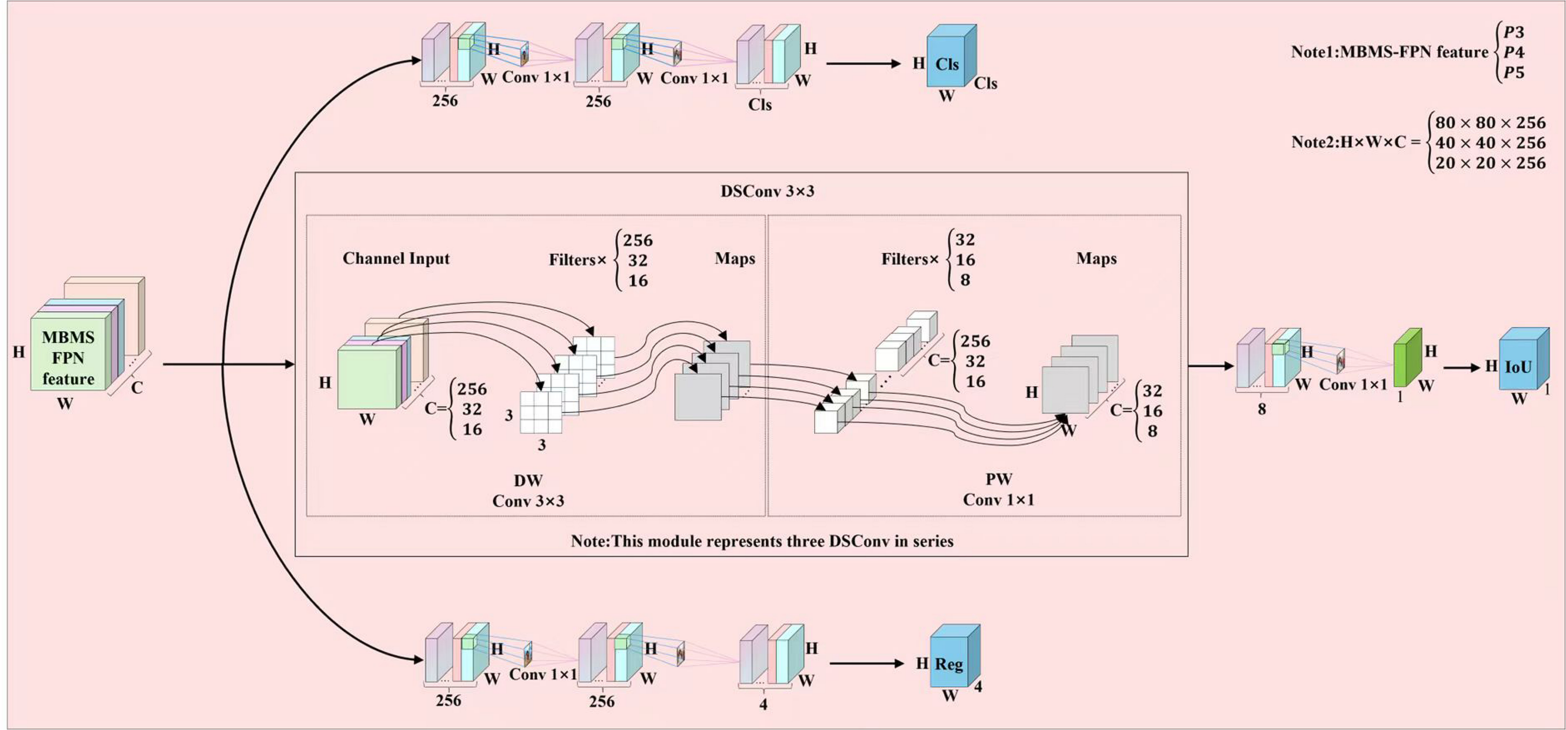
Structure of AMCCDH.

Although the detection head of YOLOv12n employs a decoupled head design, which can effectively alleviate the issue of detection accuracy degradation that arises from the conflict between regression and classification tasks in the coupled head of the original YOLO algorithm, this design significantly increases the number of model parameters. As a result, the model inference speed is slowed down, which renders it unsuitable for subsequent model deployment (Ge et al., 2021). Therefore, we improved and designed an efficient and lightweight asymmetric multi-level channel compression detection head (AMCCDH) for corn trumpets detection, which is based on the design concept of asymmetric multi-level channel compression decoupled head (Huang et al., 2024). The structure of the detection head is shown in **Figure 11**.

**Figure 12:**
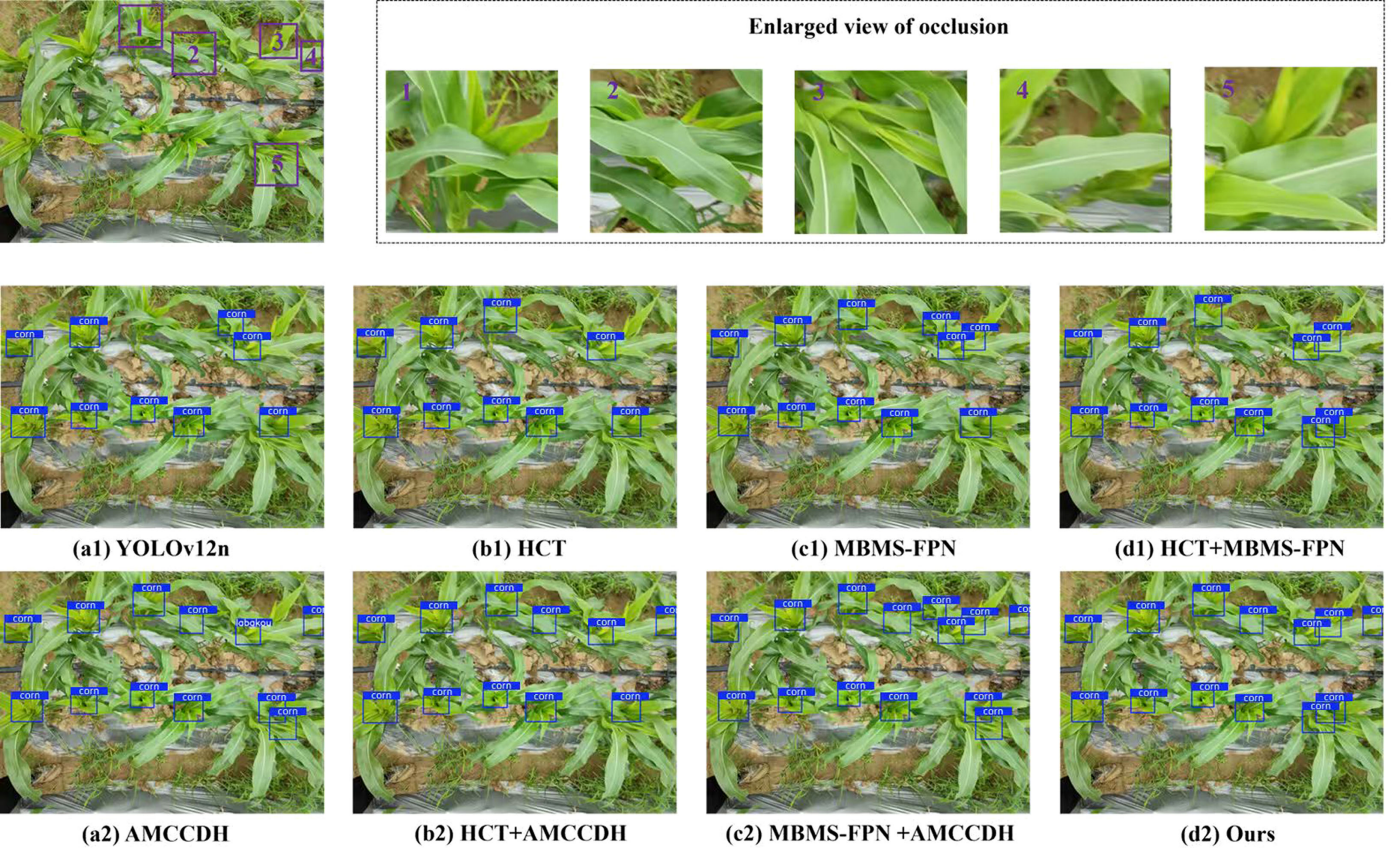
Visualization of AMCCDH application effect: **(a1)** YOLOv12n detection results; **(a2)** AMCCDH detection results; **(b1)** HCT detection results; **(b2)** HCT+AMCCDH detection results; **(c1)** MBMS-FPN detection results; **(c2)** MBMS-FPN+AMCCDH detection results; **(d1)** HCT+MBMS-FPN detection results; **(d2)** Ours' detection results.

In AMCCDH, the detector performs Cls, Reg, and IoU tasks through three different channels. In the IoU task branch, we deepen its network path and expand the receptive field of this task branch by using 3×3 depth-separable convolutions, which enables the model to better utilize contextual information, learn complex patterns in occluded scenes, and improve the accuracy of IoU predictions. Additionally, the 3×3 depth-separable convolutions split the original 3×3 convolutions in the IoU task branch into two independent steps: depth-wise convolutions and point-wise convolutions, which respectively handle spatial feature extraction and channel fusion. This approach decomposes the task and reduces redundant computations, which significantly reduces model parameters and computational complexity. The experimental results are shown in **Table 1**. By respectively comparing the 1st and 4th rows, the 2nd and 6th rows, the 3rd and 7th rows, and the 5th and 8th rows in the table, it can be seen that for any network structure, after applying AMCCDH, the accuracy, parameters, and GFLOPs of the network model are all optimized. The detection results are shown in **Figure 12**. **Figure 12**(a1-d1) shows the detection results of the models using rows 1, 2, 3, and 5 of **Table 1**, respectively, while **Figure 12** (a2-d2) shows the detection results of the models using rows 4, 6, 7, and 8 of **Table 1**, respectively. As can be seen from the figure, in **Figure 12** (a1-d1), there are corn trumpets that were not detected due to occlusion, while in **Figure 12** (a2-d2), all of them were effectively detected.

**Table 1:** Ablation experiment results.

Baseline	HCT	MBMS-FPN	AMCCDH	mAP@0.5 (%)	Precision (%)	Recall (%)	Parameters (M)	Size (MB)	FPS	GFLOPs (G)
✓				89.0	87.1	80.7	2.508	5.2	99.2	5.8
✓	✓			89.6	88.3	81.5	1.992	4.1	115.8	5.6
✓		✓		90.4	90.2	81.8	2.123	4.6	123.5	6.7
✓			✓	90.9	89.3	83.0	2.207	4.6	104.6	5.3
✓	✓	✓		90.8	88.5	83.5	1.577	3.4	126.5	6.0
✓	✓		✓	90.6	87.8	83.6	1.691	3.6	109.0	5.2
✓		✓	✓	90.8	87.8	83.5	1.953	4.3	117.1	5.8
✓	✓	✓	✓	91.5	89.8	83.7	1.407	3.1	126.9	5.1

## 3 Experimental results and analysis

### 3.1 Experimental environments

The model training in this study was based on the Ubuntu 20.04 operating system, using an RTX 4090 (24GB) graphics processing unit and an Intel(R) Xeon(R) Platinum 8352V CPU @ 2.10GHz central processing unit. The experimental code was based on CUDA 12.1, Python 3.12, and PyTorch 2.3.0. The model training hyperparameters are uniformly set as shown in **Table 2**.

**Table 2:** Training hyperparameters.

Parameters	Setpoint
epochs	300
imgsz	640×640
batch	32
optimizer	SGD
warmup_epochs	3.0
warmup_momentum	0.8
warmup_bias_lr	0.1
weight_decay	0.0005
amp	False
close_mosaic	0
Lr0	0.01
Lrf	0.01
momentum	0.937

### 3.2 Evaluation metrics

This study used multiple evaluation metrics to comprehensively evaluate the performance of the improved model, which include *mAP*, Precision, Recall, *FPS*, model parameters, model size, and floating-point operations (GFLOPs). The specific definitions are as follows:

*mAP@0.5* refers to calculating the average accuracy level of the detection model by setting a threshold of 0.5 for the intersection-over-union ratio. *mAP@0.75* refers to calculating the average accuracy level of the detection model by setting a threshold of 0.75 for the intersection-over-union ratio. *mAP@50:95* refers to the mean Average Precision calculated by evaluating the detection model across a series of intersection-over-union (IoU) thresholds. The calculation formula is Equation 1:

(1) $$mAP=\frac{1}{N}\sum_{i=1}^{N}AP_{i}$$

Where *N* is the total number of categories, and $AP_{i}$ is the average accuracy of the *i*-th category.

Precision refers to the ratio of accurately detected trumpets to the total number of targets detected in the image, which reflects the accuracy of prediction results. Recall indicates the ratio of correctly identified trumpets in the image to the total number of targets in the dataset, which reflects the coverage of the detection model. The definition formulas are Equations 2, 3:

(2) $$\text{Precision}=\frac{TP}{TP+FP}$$

(3) $$\text{Recall}=\frac{TP}{TP+FN}$$

Where *TP* indicates correctly detected positive samples, *FP* refers to false-positive samples (other objects are detected as targets), and *FN* represents false-negative samples (targets are not detected).

*FPS* denotes the number of images that a model can detect per second, which demonstrates the real-time performance of the model and serves as an important indicator for evaluating whether the model is suitable for deployment on edge devices. Notably, the *FPS* metric was measured under strictly consistent inference conditions: the precision mode was fixed as FP32, the batch size was set to 1, and pre-/post-processing steps (e.g., image preprocessing and non-maximum suppression) were excluded from the timing to eliminate the interference of implementation-specific details. To ensure the reliability of the results, the measurement protocol included a 200-iteration warm-up phase to eliminate GPU initialization and cache effects, followed by 1000 repeated inference tests to calculate the average latency. The formula for calculating FPS is Equation 4:

(4) $$FPS=\frac{1000}{\text{Total refer time}(\text{ms})}$$

The model parameters metric is defined as the total number of weight parameters to be learned in the model, the model size is defined as the disk space occupied by the weight file, and GFLOPs is used to characterize the computational complexity of the model.

### 3.3 Ablation experiment of model

The HMA-YOLO detection model for corn trumpets consists of three improved modules: HCT, MBMS-FPN, and AMCCDH. These modules are integrated into the YOLOv12n object detection model to improve the detection accuracy of corn trumpets and achieve lightweight detection. To evaluate the effectiveness of the proposed improvement method, we first conducted ablation experiments on each module, and the corresponding experimental results are shown in **Table 1**. Experimental results show that the HCT structure leverages the global feature extraction capability of the Transformer structure through partial feature channels, which improves the target detection performance of the model in complex backgrounds and reduces the computational cost of the model. Compared to the baseline model YOLOv12n, it achieves significant improvements, with improvements of 0.6%, 1.2%, 0.8%, and 16.6 s⁻¹ in mAP@0.5, Precision, Recall, and FPS, respectively. Additionally, the parameters, size, and computational complexity of the model decrease by 0.516M, 1.1MB, and 0.2G, respectively. MBMS-FPN uses EUCB to progressively upsample the feature maps of the current stage, which effectively match the dimension and resolution of the feature maps from the next skip connection. The Fusion module performs weighted fusion of features, while NHKSM extracts multi-scale features and enhances feature expression capabilities, which improves small object detection capabilities and achieves lightweight design. Compared to the baseline model, MBMS-FPN achieves significant improvements in mAP@0.5, Precision, Recall, and FPS by 1.4%, 3.1%, 1.1%, and 24.3s^-1^, respectively, and achieves significant reductions in parameters and model size to 0.385M and 0.6MB, respectively. AMCCDH improves the detection accuracy of occluded targets while reducing model parameters by deepening the network path of the IoU task branch and using 3×3 depth-separable convolutions to expand its receptive field. Compared with the baseline model, this results in increases in the mAP@0.5, Precision, Recall, and FPS of the model by 1.9%, 2.2%, 2.3%, and 5.4s⁻¹ respectively, along with decreases in the number of parameters, model size, and computational complexity by 0.301M, 0.6MB, and 0.5G respectively. Then, we added MBMS-FPN on the basis of the HCT structure, AMCCDH on the basis of the HCT structure, and AMCCDH on the basis of the MBMS-FPN structure. The results demonstrate that the performance metrics are further improved compared with those when adding a single module. Finally, by integrating the three improved modules into the baseline model, we developed detection model HMA-YOLO. Its mAP@0.5, Precision, Recall, FPS, parameters, model size, and computational complexity ultimately reached 91.5%, 89.8%, 83.7%, 126.9s⁻¹, 1.407M, 3.1MB, and 5.1G, respectively. Compared with the baseline model, the first four metrics increased by 2.5%, 2.7%, 3.0%, and 27.7 s⁻¹, while the latter three decreased by 1.101M, 2.1MB, and 0.7G, respectively. This validates the effectiveness and feasibility of our three proposed improvements to YOLOv12n for corn trumpets detection.

### 3.4 Ablation experiment of HCT structure channel allocation

To fully validate the effectiveness of allocating a certain proportion of input channels to the Transformer module within the HCT structure for global feature information extraction, we conducted comparison experiments with different channel allocation ratios. The experimental results are shown in **Table 3**. The results indicate that, whether for the P3 or P4 feature layer, when the proportion of input channels of the feature map allocated to the CNN structure is 50% or higher, the model shows either decreased detection accuracy or reduced detection speed. This is primarily attributed to inherent high computational complexity of the CNN structure and its limited receptive field. Conversely, when 75% of the channels are allocated to the Transformer structure, the model achieves the highest performance across metrics compared to other channel proportions, with mAP@0.5, Precision, Recall, and FPS reach 89.6%, 88.3%, 81.5%, and 115.8 s⁻¹ respectively. Meanwhile, the parameters and model size are reduced to 1.992M and 4.1MB, achieving a good balance between detection accuracy and model parameter efficiency.

**Table 3:** Ablation experiment results of HCT structural channel allocation.

P4_PTB (%)	P5_PTB (%)	mAP@0.5 (%)	Precision (%)	Recall (%)	Parameters (M)	Size (MB)	FPS	GFLOPs (G)
25	25	89.5	87.6	79.9	2.031	4.2	106.5	5.6
25	50	89.4	85.6	81.3	2.001	4.1	114.6	5.6
25	75	88.4	85.4	81.1	1.999	4.1	114.1	5.6
50	25	88.5	83.6	81.2	2.023	4.2	105.1	5.6
50	50	88.7	86.5	79.7	1.993	4.1	102.7	5.6
50	75	88.6	85.9	80.9	1.992	4.1	113.2	5.6
75	25	88.9	85.5	80.9	1.994	4.2	105.2	5.6
75	50	86.9	85.4	80.4	1.993	4.1	114.7	5.6
75	75	89.6	88.3	81.5	1.992	4.1	115.8	5.6

### 3.5 Comparison experiment of MBMS-FPN fixed channel dimension

Fixing the channel dimensions of the input and output feature maps of each module in MBMS-FPN can reduce model parameters and computational complexity. To evaluate the impact of fixed channel dimensions on the detection performance of the MBMS-FPN module, we used the trumpets dataset and conducted comparative experiments with different fixed channel dimensions. The experimental results are shown in **Table 4**. We observed that when the channel dimension of the feature map in MBMS-FPN is fixed at 256, the model achieves the optimal balance among the parameters, computational complexity, detection accuracy, and detection speed. While fixing the channel dimension at 128 results in the fewest parameters and computational complexity for the model, it significantly sacrifices detection accuracy. Since an increase in the channel dimension directly leads to a rise in the number of convolution kernel groups, which in turn causes parameters and computational complexity of the model to increase linearly with the channel dimension. Thus, when the channel dimension is set to 512 and 1024, such a setting does not meet our lightweight requirements for model improvement. Therefore, we determined that setting the channel dimension of the MBMS-FPN module to 256 can effectively meet the model lightweight requirements and optimize the detection accuracy of corn trumpets.

**Table 4:** Comparison experiment results of MBMS-FPN fixed channel dimension.

Head_channel	mAP@0.5 (%)	Precision (%)	Recall (%)	Parameters (M)	Size (MB)	FPS	GFLOPs (G)
128	87.7	86.2	80.4	1.914	4.3	89.8	5.7
256	90.4	90.2	81.8	2.123	4.6	123.5	6.7
512	89.6	88.6	81.4	3.514	7.3	86.3	12.2
1024	88.3	88.2	80.8	8.558	17.0	64.5	31.2

### 3.6 Comparison experiment on the size of the neck heterogeneous kernel group

Compared with the baseline, we adopt a feature map channel parallel structure and use three different convolutional kernels (depth-wise convolution based on multi-scale convolutional kernel module) to extract feature information from the current feature layer. This structure can effectively reduce parameter redundancy. However, variations in convolutional kernel sizes within the parallel structure have a significant impact on the experimental results. To verify how these kernel size differences affect the outcomes, we conducted extensive comparative experiments, including introducing parallel-structure convolutional kernels of the same scale into different feature layers, as shown in rows 2–6 of **Table 5**. Considering that different feature layers have different resolutions, we used parallel-structure convolutional kernels of different scales in different feature layers, as shown in rows 7–16 of **Table 5**. In order to further improve the feature extraction capability of the convolutional kernel for the current feature layer, we chose to use the same type of convolutional kernel in different feature layers but used multi-scale convolutional kernels in the same feature layer, as shown in rows 17–23 of **Table 5**. To adapt to the trend of varying feature layer resolutions, we employed DCMSCK in the feature layers and incrementally increased the kernel size by a step of 2 from lower to higher feature layers, as shown in rows 24–25 of **Table 5**. Experimental results show that using large convolutional kernels in different feature layers can expand the receptive field and improve accuracy, but this increases model parameters and computational complexity, which does not align with our lightweight design philosophy. Conversely, using small convolutional kernels in different feature layers can reduce model parameters and computational complexity, but their limited receptive field leads to a decrease in detection accuracy. However, when selecting heterogeneous kernel groups to extract feature layer information, we achieve an excellent balance among high accuracy, low parameters, and low computational complexity. This validates the advanced performance of the proposed neck heterogeneous kernel selection mechanism for multi-scale feature extraction, which enhances the ability of the model to detect small targets like corn trumpets and adapt to detecting corn trumpets at different heights.

**Table 5:** Comparision experimental results of the sizes of neck heterogeneous kernel.

	P3	P4	P5	mAP@0.5 (%)	Precision (%)	Recall (%)	Parameters (M)	Size (MB)	FPS	GFLOPs (G)
Baseline	3×3	3×3	3×3	89.0	87.1	80.7	2.508	5.2	99.2	5.8
Ablation	1、1、1	1、1、1	1、1、1	88.7	85.4	80.1	2.056	4.5	60.4	6.5
	3、3、3	3、3、3	3、3、3	89.1	86.1	81.2	2.074	4.5	61.0	6.6
	5、5、5	5、5、5	5、5、5	89.2	87.5	82.3	2.111	4.6	59.5	6.9
	7、7、7	7、7、7	7、7、7	90.3	87.2	81.9	3.166	4.7	62.5	7.2
	9、9、9	9、9、9	9、9、9	90.3	87.9	83.1	2.241	4.8	70.7	7.6
	1、1、1	3、3、3	5、5、5	90.1	87.0	80.8	2.080	4.5	58.5	6.6
	1、1、1	3、3、3	7、7、7	90.4	87.5	81.6	2.099	4.5	68.0	6.6
	1、1、1	3、3、3	9、9、9	90.5	88.2	81.8	2.123	4.6	57.7	6.7
	1、1、1	5、5、5	7、7、7	89.8	87.9	80.1	2.111	4.6	59.2	6.6
	1、1、1	5、5、5	9、9、9	90.3	88.9	81.6	2.136	4.6	55.8	6.7
	1、1、1	7、7、7	9、9、9	90.3	87.6	82.5	2.154	4.6	60.6	6.7
	3、3、3	5、5、5	7、7、7	90.2	86.4	79.0	2.117	4.6	60.5	6.7
	3、3、3	5、5、5	9、9、9	90.4	87.4	83.1	2.141	4.6	62.2	6.7
	3、3、3	7、7、7	9、9、9	90.5	88.5	81.2	2.160	4.7	62.2	6.8
	5、5、5	7、7、7	9、9、9	89.7	85.9	81.9	2.173	4.7	68.9	6.9
	5、7、9	5、7、9	5、7、9	90.1	87.9	81.4	2.173	4.7	59.4	7.2
	3、5、7	3、5、7	3、5、7	90.3	87.3	81.6	2.118	4.6	62.9	6.9
	1、3、5	1、3、5	1、3、5	89.5	88.9	81.4	2.080	4.5	59.8	6.7
	1、3、5	1、3、5	3、5、7	88.6	86.2	82.7	2.093	4.5	100.3	6.7
	1、3、5	1、3、5	5、7、9	90.6	88.1	81.1	2.111	4.6	103.8	6.7
	1、3、5	3、5、7	3、5、7	89.2	85.4	82.1	2.105	4.6	103.0	6.7
	1、3、5	5、7、9	5、7、9	89.8	88.2	82.0	2.142	4.6	105.1	6.8
	3、5、7	3、5、7	5、7、9	90.1	85.8	82.3	2.137	4.6	109.54	6.9
	3、5、7	5、7、9	5、7、9	90.3	88.9	81.2	2.154	4.6	117.6	7.0
Ours	1、3、5	3、5、7	5、7、9	90.4	90.2	81.8	2.123	4.6	123.5	6.7

### 3.7 Comparison experiments with other detection models

To comprehensively evaluate the performance of the proposed HMA-YOLO detection model, we conducted a series of comparative experiments on various mainstream object detection models, including one-stage and two-stage models. The comparison experiments were conducted under the same training parameters on the corn trumpets dataset to ensure fair evaluation across models. **Table 6** provides a comprehensive overview of the metrics used to evaluate model performance, including mAP@0.5, Precision, Recall, Parameters, Model size, GFLOPs, and FPS. The experimental results indicate that among various mainstream detection models, although RT-DETR-X and RT-DETR-R18 achieved relatively high mAP@0.5 scores of 90.9% and 91.4%, respectively, they have a high number of parameters and high complexity, which is attributed to the global modeling capabilities and end-to-end design of the underlying Transformer architecture. Similarly, YOLOv7-tiny retains a large number of convolutional layers, residual modules, and multi-scale detection branches to improve detection accuracy, which results in a relatively high parameter count and complexity. Thus, the aforementioned three models are not suitable for deployment on edge devices with limited computational resources. However, YOLOv12n introduces a regional attention module, a residual effective layer aggregation network, and an upgraded attention-centric architecture, which achieves a good balance between detection accuracy and computational complexity. Therefore, this paper selects YOLOv12n as the baseline model and clearly defines the improvement strategy as maximizing detection accuracy for corn trumpet targets while further lightweighting the model.

**Table 6:** Comparison experiments results of different object detection models.

Model	mAP@0.5 (%)	Precision (%)	Recall (%)	Parameters (M)	Size (MB)	GFLOPs (G)	FPS
SSD	81.5	80.4	78.7	24.280	94.2	62.7	127.1
Faster-RCNN	71.4	62.5	63.5	29.138	132.7	474.1	100.6
YOLOv5n	80.5	78.8	72.3	2.503	5.0	7.1	100.8
YOLOv7-tiny	90.1	88.9	81.4	6.015	12.3	13.2	84.7
YOLOv8n	81.2	77.9	72.1	3.005	5.9	6.4	87.5
YOLOv9t	88.6	85.4	78.9	2.612	6.1	10.7	100.6
YOLOv10n	88.3	85.9	79.3	2.265	5.5	6.5	117.3
YOLOv11n	88.6	87.9	79.5	2.582	5.2	6.3	78.5
YOLOv12n	89.0	87.1	80.7	2.508	5.2	5.8	99.2
Hyper-YOLO	86.8	85.9	79.9	3.942	7.8	10.8	97.4
RT-DETR-X	90.9	85.6	86.7	65.470	257.7	222.5	88.7
RT-DETR-R18	91.4	88.4	84.1	19.873	76.9	56.9	135.96
Ours	91.5	89.8	83.7	1.407	3.1	5.1	126.9

We introduced HCT into the backbone network of the YOLOv12n model, replaced the neck with MBMS-FPN, and used AMCCDH, which achieves the highest mAP@0.5 and Precision scores of 91.5% and 89.8%, respectively. The model also demonstrates superior performance in the recall metric with a score of 83.7%. In terms of computational efficiency, compared to the baseline model, our improved model significantly reduced GFLOPs by 12%, with model parameter count and size reduced to 1.407M and 3.1M, respectively. The FPS metric further highlighted the efficiency of the model, which achieves a frame rate of 126.9 s^-1^ and enables the improved model to effectively accommodate real-time application requirements in resource-constrained environments.

### 3.8 Performance verification of occlusion, small object, and background similarity scenarios under strict IoU thresholds

To verify the detection robustness of the proposed model under core challenging scenarios (i.e., occlusion, small objects, and background similarity), a performance evaluation experiment with stricter IoU thresholds is conducted in this study. Two key metrics are adopted, namely mAP@0.75 (mean Average Precision with an IoU threshold of 0.75) and mAP@50:95 (mean Average Precision computed across IoU thresholds from 0.5 to 0.95 at a step size of 0.05). These two metrics respectively focus on the detection capability under high localization accuracy requirements and the performance stability across multiple IoU thresholds. Experimental results are presented in **Table 7**. As shown in **Table 7**, mAP@0.75, a metric that quantifies refined localization accuracy, yields a 7.7% improvement for HMA-YOLO in comparison with the Baseline. This enhancement demonstrates that HMA-YOLO can effectively increase the overlap between the predicted bounding boxes of small objects and the ground truth boxes while alleviating localization deviations in scenarios with similar backgrounds, and thus it specifically addresses the core challenge of insufficient localization accuracy. Similarly, mAP@50:95, a metric that reflects performance stability across varying IoU thresholds, achieves a 5.4% improvement for HMA-YOLO over the Baseline. This result confirms that HMA-YOLO can maintain stable detection performance regardless of whether the localization requirements are loose or stringent. The model also presents a significantly smaller amplitude of performance degradation than the Baseline in challenging scenarios corresponding to high IoU thresholds, such as occlusion and complex backgrounds, which further validates the strong robustness of the model under core challenging conditions.

**Table 7:** Experimental results of model performance evaluation under strict IoU thresholds.

Model	mAP@.75(%)	mAP@50-95(%)
Baseline	36.0	43.8
HMA-YOLO	43.7	49.2

### 3.9 Visualization of detection results

**Figure 13:**
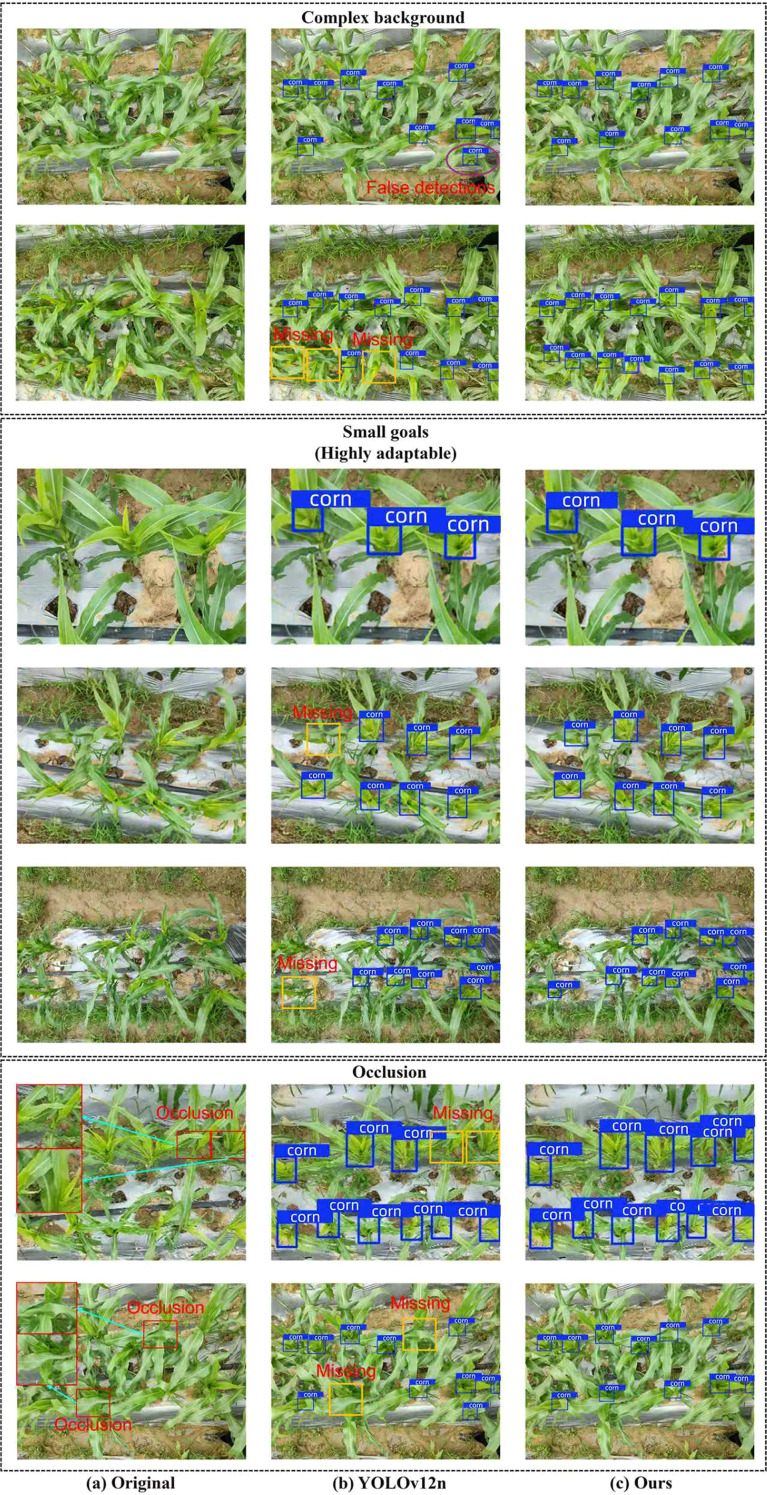
Visualization of detection: **(a)** Three types of original images representative of the characteristics of the dataset; **(b)** YOLOv12n detection results; **(c)** Ours' detection results.

To validate the advantages of the model proposed in this study over the benchmark model YOLOv12n in detecting corn trumpets, we randomly selected several images from the test set that were representative of the dataset characteristics for visual detection, including targets with similar colors to the background, small target sizes, and targets obscured by corn leaves, as shown in **Figure 13**. The comparison results indicate that the detection performance of the baseline model is below the standard detection performance level, primarily due to false positives and false negatives. The three primary factors accounting for false negatives in the baseline model are: high color similarity between corn trumpets and the background, which makes it difficult for the model to effectively identify the target from the background; structural obstruction of the corn trumpets by corn leaves; and the small size of the corn trumpets (coupled with poor adaptability of the visual detector to variations in corn trumpet height). These issues are illustrated by the orange rectangular boxes in the figure. In addition, the fact that the color of the corn trumpets is highly similar to the background color can cause the baseline model to misidentify corn leaves as corn trumpets, which results in misdetection, as shown in the purple oval frame in the figure. In contrast, the improved model proposed in this study for YOLOv12n demonstrates significant improvements in addressing the aforementioned issues. During object detection, the NHKSM in MBMS-FPN, HCT in the backbone, and AMCCDH all effectively mitigate false negatives, and objects that were previously missed are now successfully identified within the orange rectangular bounding boxes. Additionally, HCT effectively addresses false positives, accurately excluding leaves within the purple elliptical box from the detection results.

### 3.10 Fault case and generalization risk analysis

**Figure 14:**
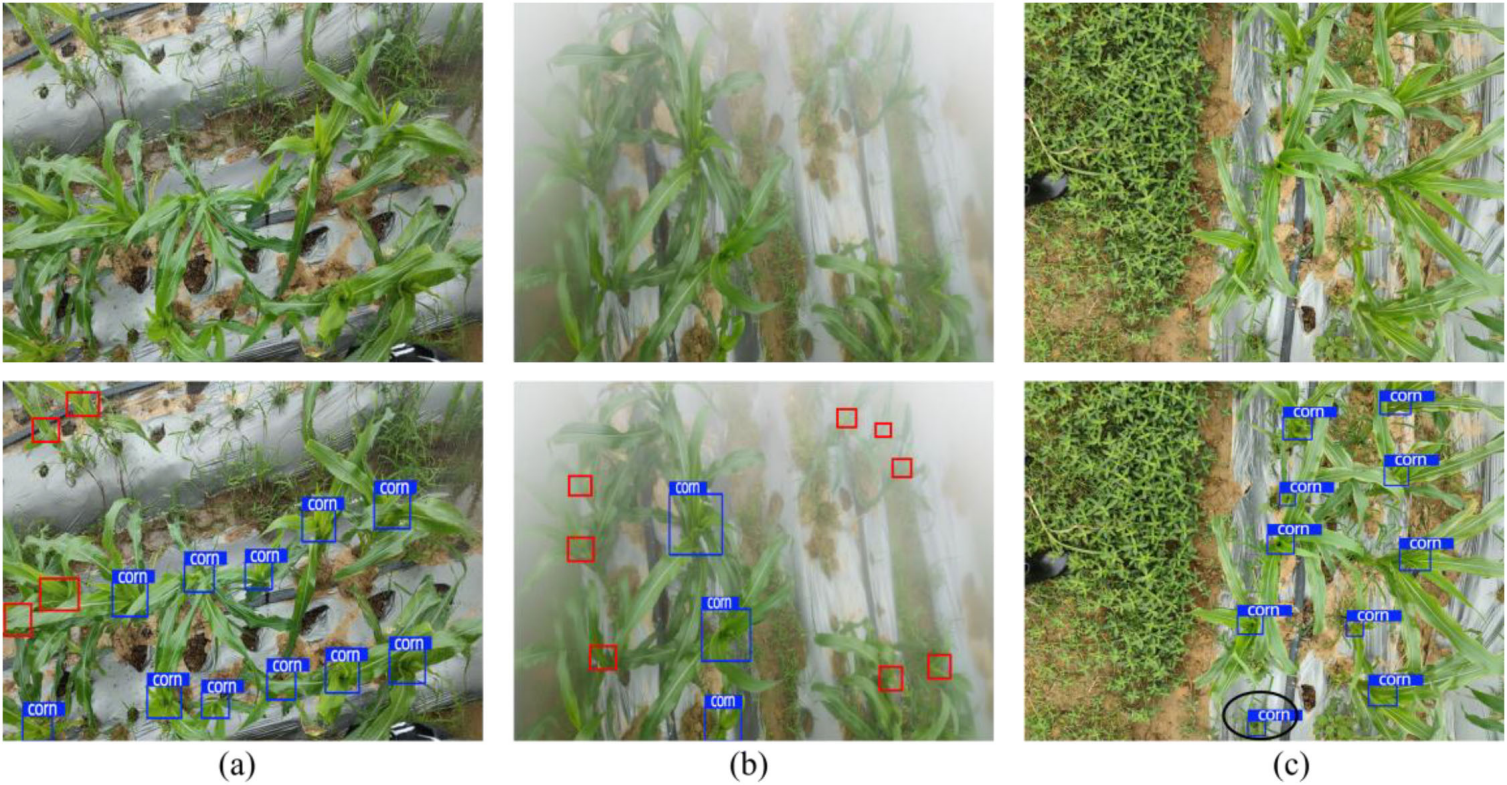
Fault case display: **(a)** Detection results under rainy weather conditions; **(b)** Detection results under foggy weather conditions; **(c)** Detection results under the condition of similar object interference.

Although the HMA-YOLO model demonstrates excellent accuracy, efficiency, and lightweight advantages in the task of corn trumpets detection, it still has certain limitations and generalization risks when combined with actual application scenarios. In terms of performance in fault scenarios, due to the limited coverage of the dataset, the model has not been trained under adverse weather conditions such as rain and fog. In such scenarios, the model is prone to an increase in the rate of missed detections due to blurred target features and enhanced background interference, as shown in the red rectangular boxes in (**Figures 14a, b**). Additionally, the corn trumpets is easily confused with similar objects in the field, such as young weeds, and the interference from such similar objects can cause the model to misidentify non-target objects as detection targets, thereby increasing the false detection rate, as shown in the black elliptical box in **Figure 14c**).

The core causes of these limitations include: first, data distribution bias, as the dataset lacks coverage of extreme weather samples, which results in the model not learning the feature patterns of such scenarios; second, insufficient model structure adaptability, as the existing model has limited ability to capture the features of extreme scenarios and atypical targets. From the perspective of generalization risk, when the model is transferred to different regional farmland environments, its performance may deteriorate due to significant differences in climate and soil texture among different production areas.

Future research will focus on targeted optimization efforts: first, expanding the dataset by supplementing labeled samples under adverse weather conditions to improve the completeness of data distribution; second, iterating the model structure to optimize its adaptability and enhance the detection performance for extreme scenarios and atypical targets.

### 3.11 Deploying the HMA-YOLO model on NVIDIA Jetson Xavier NX

NVIDIA Jetson Xavier NX is an embedded device designed for artificial intelligence and deep learning applications. Its compact design, powerful computing performance, and low power consumption enable it to be deployed in a variety of environments and run for long periods of time, which meets the needs of continuous system operation (Dong et al., 2025). Therefore, the device was employed by this study as the deployment platform for the HMA-YOLO model, which aims to verify the real-time detection effectiveness of the HMA-YOLO model on edge devices and facilitate the rapid identification of corn trumpets by the precision application system. **Table 8** shows the detailed information about the model deployment environment.

**Table 8:** Hardware and environment information for edge device deployments.

Parameters	Setpoint
Power dissipation	5W - 10W
GPU	384 CUDA cores, 48 Tensor Cores and 2 NVDLA
CPU	6-core NVIDIA Carmel ARM^®^v8.2 64-bit CPU 6 MB L2 + 4 MB L3
Operating system	Ubuntu20.04
Python	3.8.20
CUDA	11.4
CUDNN	8.6.0
PyTorch	2.0.0NV23.05
Torchvision	0.15.1

**Figure 15:**
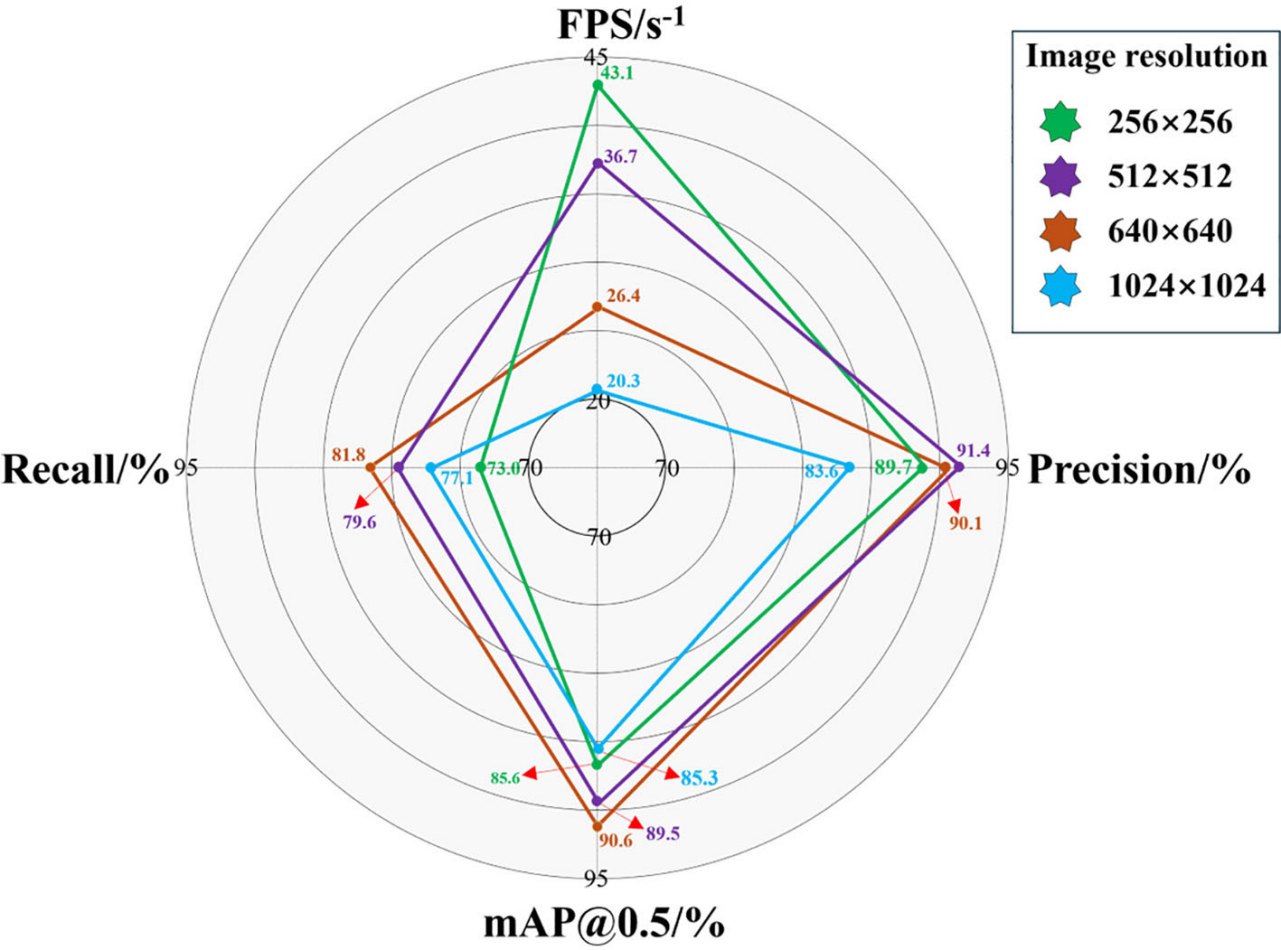
Differences in precision and speed indicators of HMA-YOLO on edge devices under different image input sizes.

Subsequently, we investigated the impact of four different input image resolutions on the accuracy and speed of the HMA-YOLO model in edge device deployment scenarios, as shown in **Figure 15**. The experimental results show that within the input image resolution range from 256×256 to 640×640, both the recall and mAP@0.5 of the model increase with increasing resolution. This is because the model can extract more detailed feature information from larger input image resolutions, which thereby improves detection accuracy. However, recall, mAP@0.5 and precision show a downward trend when the image resolution reaches 1024×1024. This may be because edge computing devices have limited hardware resources, and processing high-resolution input images increases the computational burden, which in turn degrades the detection performance of the model. At the same time, we also observed that the FPS shows a significant downward trend as the resolution of the input image increases. This is mainly because larger image resolutions have more pixels for the model to process, which increases the computational load on the device and slows down the inference speed. Therefore, to achieve the optimal balance between detection speed and accuracy, we ultimately selected 640×640 as the input image resolution.

**Figure 16:**
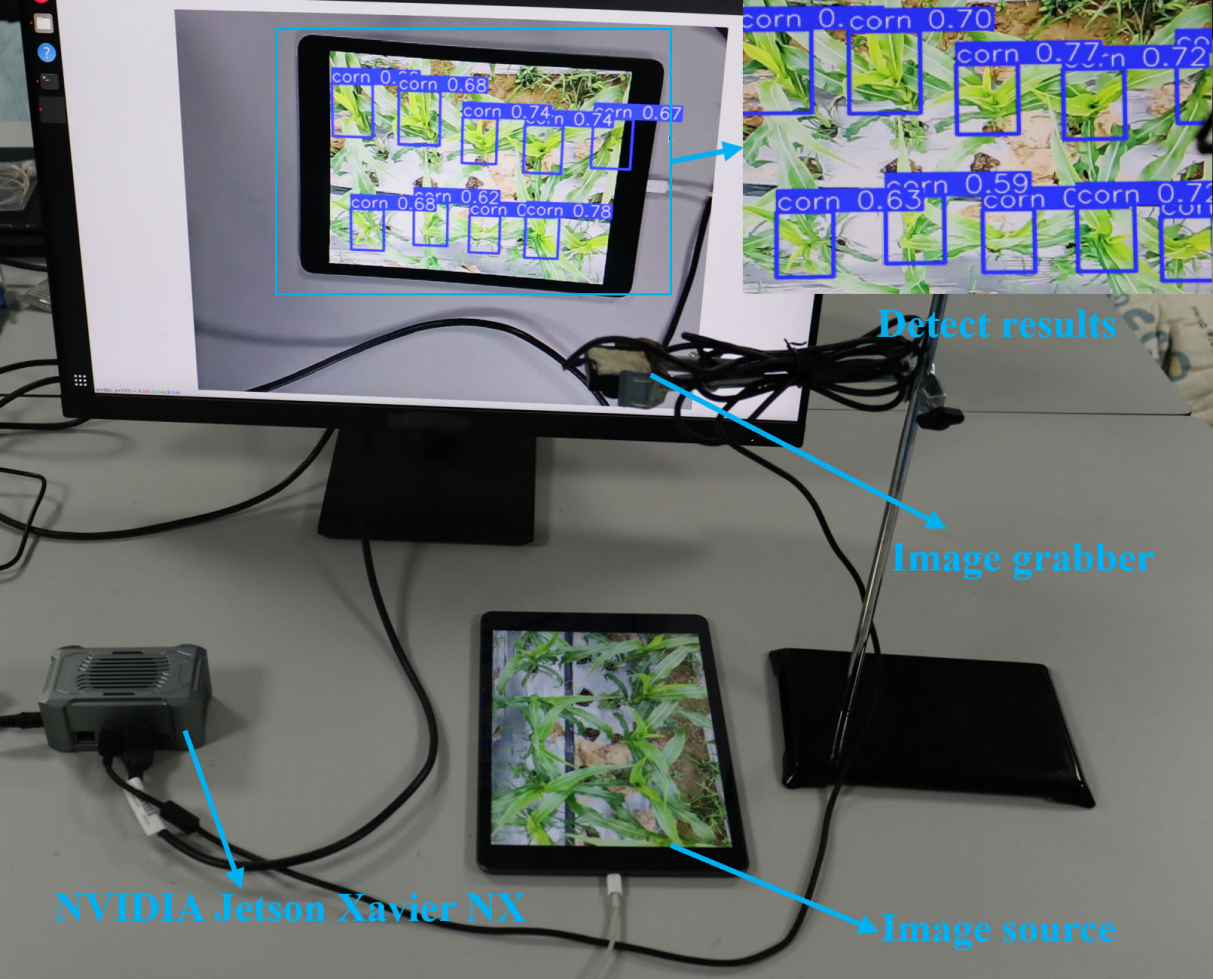
Detection performance of HMA-YOLO on edge devices.

The HMA-YOLO model was deployed on the NVIDIA Jetson Xavier NX device to verify its actual detection performance on edge devices, with the results shown in **Figure 16**. Excellent detection performance is exhibited by the HMA-YOLO model in images of corn trumpets, which can effectively identify targets, though occasional missed or false detections may occur. This indicates that the model can still accurately and rapidly accomplish detection tasks in edge environments with limited computing power.

## 4 Discussion

This paper proposes an effective detection model for corn trumpets in scenarios with limited computing resources, which we name HMA-YOLO model. It provides reliable visual support for the efficient operation of corn precision spraying systems. The model is based on YOLOv12n and has been optimized in terms of the backbone network, neck network, and head network. Extensive experiments have demonstrated that the HMA-YOLO model outperforms mainstream object detectors in terms of accuracy and computational efficiency, enabling it to accurately detect corn trumpets with excellent robustness.

Although the model performs well in terms of multiple performance evaluation metrics, the issue of missed detections has not been fully resolved, which results in suboptimal recall rates and room for improvement. Additionally, since we only collected data on three key states of the corn trumpets under clear weather conditions, we did not consider the potential interference that rainy, foggy weather conditions, diverse soil backgrounds, and different corn varieties might have on the detection results of the model. To address these challenges, in future work we plan to further enhance the feature extraction capabilities of the model through algorithm optimization and introduce more diverse data samples to cover a wider range of weather, lighting, and field conditions, aiming to significantly improve the detection robustness and the generalization ability of the model. Meanwhile, in future research, we intend to combine RGB images with infrared images using a bimodal approach to enhance the ability of the model to identify corn trumpets in complex backgrounds, and carry out cross-domain validation by extending the proposed method to the identification scenarios of different crop growth stages, so as to further verify the cross-scenario adaptability and practical application value of the model.

## 5 Conclusion

To enable the precision application system to accurately identify corn trumpets in complex farmland environments, we proposed the HMA-YOLO model, which is based on the YOLOv12n network. The main results are summarized as follows:

1. To enhance the detection accuracy and efficiency of corn trumpets, we made improvements to YOLOv12n in three aspects and proposed the HMA-YOLO model. First, we replaced the A2C2f module of the backbone network with an HCT structure to improve the ability of the model to perceive corn trumpets in complex contexts. Secondly, the neck network was replaced with MBMS-FPN to enhance the detection accuracy of the model for small targets such as corn trumpets and improve the adaptability of the model to changes in trumpet height during the detection process, which primarily extracts and fuses deep target feature information through the neck heterogeneous kernel selection mechanism and weighted feature fusion module. Finally, the AMCCDH was used as the detection head, which effectively improves the detection accuracy for occluded targets. Furthermore, all three improvements have been optimized for weight reduction.
2. Experimental results on the enhanced corn trumpets dataset indicate that the HMA-YOLO model achieves an mAP@0.5 of 91.5%, precision of 89.8% and recall of 83.7%, which outperform other mainstream object detectors. Additionally, the processing speed is improved from 99.2 s^-1^ in the baseline model to 126.9 s^-1^, while the parameters, model size, and GFLOPs are only 1.407M, 3.1MB, and 5.1G, respectively. These parameters demonstrate that the HMA-YOLO model can detect corn trumpets in real time with high accuracy in environments with limited computing resources.
3. We successfully deployed the HMA-YOLO model on NVIDIA Jetson Xavier NX, an embedded edge device, which proves the feasibility and robustness in practical application scenarios.

In summary, the HMA-YOLO model proposed in this study effectively meets the requirements of detection accuracy and real-time performance, which makes it suitable for deployment in a precision application visual system for corn trumpets detection. This will help improve application efficiency and reduce pesticide waste, which provides key technical support for the prevention and control of corn pests and diseases in the context of smart agriculture.

## Data Availability

The original contributions presented in the study are included in the article/supplementary material. Further inquiries can be directed to the corresponding authors.
